# Inter-sexual mate competition in three cultures

**DOI:** 10.1371/journal.pone.0236549

**Published:** 2020-07-29

**Authors:** Scott W. Semenyna, Francisco R. Gómez Jiménez, Doug P. VanderLaan, Paul L. Vasey

**Affiliations:** 1 Department of Psychology, University of Lethbridge, Lethbridge, Canada; 2 Department of Psychology, University of Toronto Mississauga, Mississauga, Canada; 3 Child and Youth Psychiatry, Centre for Addiction and Mental Health, Toronto, Canada; University of Wroclaw, POLAND

## Abstract

Darwinian sexual selection theory holds that mate selection occurs inter-sexually, and mate competition occurs intra*-*sexually for opposite-sex partners. We demonstrate that *inter*-sexual mate competition can also occur among humans at appreciable rates that vary by culture. In Canada, inter-sexual mate competition was both rare and inconsequential. However, data from two disparate non-Western cultures—Samoa and the Istmo Zapotec (Oaxaca, Mexico)—show that women frequently compete with feminine same-sex attracted males to acquire and maintain masculine male mates (i.e., men). Inter-sexual mate competition most commonly involved feminine males attempting to poach women’s masculine male sexual partners. During these interactions, women and feminine males both attempted to manipulate the man who was the object of sexual competition; feminine males attempted to entice the target man, whereas women engaged in guarding and emotionally punitive behaviours. We do not anticipate that inter-sexual mate competition will be common in most species or across all cultures. However, when males and females prefer the same sexual partners, who themselves behave in a bisexual manner, then inter-sexual mate competition can ensue.

## Introduction

A core tenet of Darwinian sexual selection theory is that mate selection occurs inter-sexually, and competition occurs intra*-*sexually for opposite-sex partners, a pattern that has been documented in most species [1]. Sexual selection is known to favor individuals who demonstrate flexibility in the means by which they compete for reproductive partners [2, 3], and varied intra-sexual mate competition tactics are both widespread and well documented [4–7]. In this study, we demonstrate that when males and females prefer the same sexual partners, who themselves behave in a bisexual manner, then *inter*-sexual mate competition can ensue. Quantitative study of inter-sexual mate competition exists for only one species, Japanese macaques (*Macaca fuscata*), in which males and females compete for female sexual partners [8]. Anecdotal evidence suggest that males and females compete inter-sexually for sexual partners in at least fourteen additional non-human species across eight different avian and mammalian Orders, and ethnographic anecdotes suggest that inter-sexual mate competition also occurs in humans [9].

Gender complicates discussions of inter-sexual mate competition in humans because same-sex sexual interactions can be structured in a manner that is *homogendered* (i.e., partners occupy the same gender category) or *heterogendered* (i.e., partners occupy different gender categories) [10]. In the contemporary West, same-sex sexual interactions are usually homogendered, typically occurring between two “gay” men or two “lesbian” women [11]. However, in many non-Western cultural contexts, same-sex interactions are heterogendered, because they occur between a cisgender individual and a partner who occupies a culturally-recognized gender category that exists beyond the “man” or “woman” binary [10, 12, 13]. In particular, feminine biological males in many non-Western cultures are not seen as “men” nor “women,” but rather as a non-binary third gender [12, 14]. Examples of feminine, non-binary males include the *bissu* of Sulawesi, the *hijra* of India, the *xanith* of Oman, and the *‘yan dandu* of Nigeria [10]. From the perspective of psychobiology, the most well studied third-gender males are the Samoan *fa’afafine* [15] and the Istmo Zapotec *muxes* of Oaxaca, Mexico [16]. These individuals are almost always exclusively androphilic (i.e., sexually attracted and aroused to adult males), and display the same correlates of male androphilia documented among Western gay men, such as gender atypicality in childhood, more older brothers, more androphilic relatives, and a prevalence rate of ~2–5% [14, 17, 18]. Gender non-binary males such as these rarely, if ever, engage in sexual activity with each other, but instead engage in sexual activity with masculine males (i.e., men). Most of the men who engage in sexual activity with *fa’afafine* and *muxes* also engage in sexual activity with women, preferring the latter as sexual partners [16, 19]. Although male androphilia does not vary appreciably across historical [20] or cultural [21, 22] contexts (i.e., <5% of males), gynephilic men’s willingness to engage in sexual activity with feminine males varies considerably across different cultural milieus [23, 24]. Importantly, in both Samoa and among the Istmo Zapotec, there is widespread cultural tolerance, and in certain contexts even celebration, of *fa’afafine* [25] and *muxes* [26].

A number of ethnographic accounts suggest that inter-sexual mate competition occurs between women and gender non-binary males. For example, a Mohave gender non-binary male (*alyha*) incited a fight against the wife of a man who was a former lover, with the man interceding to prevent a physical altercation between his new bride and the *alyha* [27]. Similar tension has been described between Javanese women and gender non-binary males (*banci*) who attempt to seduce their husbands [28]. Presently, we sought to move beyond anecdotal accounts of human inter-sexual mate competition, and instead document the phenomenon more systematically in three distinct cultures: Canada, Samoa, and among the Istmo Zapotec of Oaxaca, Mexico.

First, we aimed to document the frequency of inter-sexual mate competition in these three cultures. Because it is virtually certain that most *fa’afafine* and *muxe* have engaged inter-sexual mate competition, data collection focused on the experiences of women, who also represent the reproductive competitors most relevant to sexual selection. We predicted that Canadian women’s experiences of inter-sexual mate competition would be relatively rare because male bisexuality occurs infrequently in Western cultural contexts [11] and heterosexual men are largely disinterested in same-sex contact with gay men. In contrast, we predicted that Samoan and Istmo Zapotec women would report relatively high levels of inter-sexual mate competition because both *fa’afafine* and *muxes* routinely engage in sexual activity with masculine men who behave bisexually. Second, we endeavored to document the features of inter-sexual mate competition when it occurs, applying established mate-competition tactics taxonomies [e.g., 4, 5, 6, 29, 30] to qualitative accounts of such interactions. Given the anticipated rarity of inter-sexual mate competition in Canada, we expected that women would dismiss such competition as trivial when it did occur, and engage in few mate-competition tactics. Conversely, we predicted that both Samoan and Istmo Zapotec women would perceive inter-sexual competition as a genuine threat, engaging in mate attraction tactics (e.g., flirting, appearance enhancement) during acquisition contests, and mate-retention strategies (e.g., guarding and relationship-manipulation) when *fa’afafine* or *muxe* attempted to poach their mates.

## Method

All study measures were approved by the office of research ethics at the University of Lethbridge. A Samoan research visa was obtained from the Samoa Immigration Office with the support of the Samoan *Fa’afafine* Association. Canadian foreign nationals, and US Citizens, are permitted to conduct research in Mexico for a period of 180 days if they have a valid passport [31]. In addition, however, we obtained a letter endorsing our research from the Office of the Municipal President in Juchitán, Mexico. All participants signed informed consent forms before participation, and completed basic biodemographic measures including age, relationship status, and a 7-point Kinsey-style sexual orientation measure [32].

### Participants

Data were collected from three Canadian sub-samples of women, two from undergraduate research pools (*n* = 138 and 144 respectively), and a community sample of non-student participants from a pub and eatery in a small city in Southern Alberta, Canada (*n* = 62). The pooled Canadian sample (*n* = 344) had an average age of 25.10 years (*SD*_age_ = 11.68, range 17–70), with most women (76.7%) in committed romantic relationships with men for a median of 2 years among undergraduates, and ≥10 years among the community sample. The Canadian sample was comprised primarily of Caucasian women (80.2%) with the remainder including other ethnicities (primarily South Asian, East Asian, Canadian First Nations, or “other”). The majority of Canadian women were exclusively or mostly heterosexual (93.9%), with the remainder reporting some attraction to women (i.e., Kinsey 2–4, all of whom were in relationships with men). We interviewed Samoan women (*n* = 128) on the island of Upolu in the Independent State of Samoa, a Polynesian island nation in the South Pacific. The average age of the Samoan women was 33.96 years (*SD*_age_ = 11.7, range 19–70). Most women were in heterosexual relationships (26 dating, 89 married), although some (*n* = 13) were single. Nearly all Samoan women were exclusively heterosexual (Kinsey 0), although one woman was bisexual (Kinsey 3). We also interviewed indigenous Zapotec women (*n* = 100) in the Istmo region (Juchitán and Tehuantepec districts) of Oaxaca, Mexico. The average age of the Istmo Zapotec women was 32.55 (*SD*_age_ = 10.6, range 18–70). Most were in heterosexual relationships (18 dating, 59 married), but some (*n* = 23) were single. Most were exclusively attracted to men (*n* = 93), but some were mostly heterosexual (Kinsey 1; *n* = 6) and one was bisexual (Kinsey 3).

Women were asked whether they had ever experienced a situation in which they and a gay man, *fa’afafine*, or *muxe* (depending on culture) were simultaneously interested in, and competed for, the romantic/sexual attention of the same man. Participants who answered in the affirmative were then asked to provide detailed information about how the interaction unfolded. Interviewers sought clarification as necessary in order to ascertain details of the event, or specific tactics employed by the participants or their rivals, but participants otherwise gave free responses.

### Treatment of data

All narrative recounts of mate-competition interactions were audio recorded, and later transcribed and translated (when necessary). Each narrative was coded by two independent raters using a psychometrically validated mate retention inventory taxonomy [4, 6, 33]. Each interaction was coded by the first author, as well as a research assistant trained in applying the mate competition taxonomy but blind to study hypotheses. Inter-rater reliability was high (Canada: 95.24±3.53% agreement, mean Cohen’s *Kappa* = .76, *SD* = 0.17; Samoa: 92.86±4.23% agreement, mean Cohen’s *Kappa* = .69, *SD* = .18; Istmo Zapotec: 92.86±4.76% agreement, mean Cohen’s *Kappa* = .69, *SD* = .18) and disagreements were resolved via discussion between the raters until consensus was reached. Tactics were coded as either present or absent for each competitor in an interaction. Cumulative frequencies were then calculated, and the presence/absence of each tactic compared between women and male competitors using 2X2 contingency tables. Due to the large number of statistical comparisons presented in Tables 1–4, attempts were made to balance Type I and Type II errors by treating Fisher’s Exact [34] two-tailed *p*-values as suggestive if they fell below *p* = .05, and significant only if they fell below *p* = .005 [35].

**Table 1:** Inter-sexual mate competition retention/poaching tactics in Canada.

	Percentage Reported	
	Female Participant (*n* = 19)	Male Rival (*n* = 19)
**Target Manipulation**	47.4	94.7*
**Guarding**	36.8	5.3^†^
Direct Guarding	5.3	0
Vigilance	31.6	0^†^
Concealment	10.5	0
Monopolize	21.1	5.3
**Negative Inducements**	15.8	10.5
Jealousy Induction	0	0
Punish Infidelity Threat	5.3	0
Emotional Manipulation	5.3	0
Commitment Manipulation	15.8	5.3
Derogate Competitor	0	5.3
**Positive Inducements**	15.8	89.5*
Resource Display	0	10.5
Sexual Inducement	5.3	31.6
Appearance Enhancement	0	0
Love and Caring	0	10.5
Submission & Debasement	0	0
Direct Contact	5.3	63.2*
Personality Advertisement	5.3	10.5
**Gain Access**	0	36.8^†^
**Competitor Manipulation**	15.8	10.5
**Signal Possession**	10.5	0
Verbal Possession Signals	5.3	0
Physical Possession Signals	5.3	0
Possessive Ornamentation	0	0
**Negative Inducements**	10.5	10.5
Derogate Target	0	0
Threated Competitor	5.3	1
Violence against Rival	0	0
Direct Action	5.3	10.5
**Capitalize on Opportunity**	0	10.5
**Coalitional Strategy**	0	0
**Strategy Matching**	5.3	0

Global competitive strategies are bold, beneath which individual competitive tactics are listed.

† *p* < .05

* (*p* < .005) indicates a significant difference between the competitive strategies or tactics employed by competitors.

**Table 2:** Inter-sexual mate competition retention/poaching tactics in Samoa.

	Percentage Reported	
	Female Participant (*n* = 41)	*Fa’afafine* Rival (*n* = 41)
**Target Manipulation**	90.2	92.3
**Guarding**	53.7	12.2*
Direct Guarding	4.9	0
Vigilance	41.5	4.9*
Concealment	14.6	4.9
Monopolize	7.3	4.9
**Negative Inducements**	70.7	2.4*
Jealousy Induction	2.4	0
Punish Infidelity Threat	43.9	0*
Emotional Manipulation	39.0	0*
Commitment Manipulation	22.0	0*
Derogate Competitor	2.4	2.4
**Positive Inducements**	24.4	87.8*
Resource Display	4.9	26.8
Sexual Inducement	2.4	46.3*
Appearance Enhancement	9.8	17.1
Love and Caring	12.2	7.3
Submission & Debasement	2.4	0
Direct Contact	4.9	56.1*
Personality Advertisement	0	2.4
**Gain Access**	0	29.3*
**Competitor Manipulation**	34.1	39.0
**Signal Possession**	24.4	26.8
Verbal Possession Signals	19.5	14.6
Physical Possession Signals	4.9	14.6
Possessive Ornamentation	0	0
**Negative Inducements**	19.5	22.0
Derogate Target	0	4.9
Threated Competitor	7.3	9.8
Violence against Rival	4.9	9.8
Direct Action	14.6	7.3
**Capitalize on Opportunity**	0	24.4*
**Coalitional Strategy**	31.7	7.3
**Strategy Matching**	9.8	9.8

Global competitive strategies are bold, beneath which individual competitive tactics are listed.

* (*p* < .005) indicates a significant difference between the competitive strategies or tactics employed by competitors.

**Table 3:** Inter-sexual mate acquisition competition tactics in the Istmo Zapotec.

	Percentage Reported	
	Female Participant (*n* = 30)	*Muxe* Rival (*n* = 30)
**Target Manipulation**	80.0	96.7
**Guarding**	16.7	20
Direct Guarding	0	3.3
Vigilance	10.0	6.7
Concealment	6.7	3.3
Monopolize	0	10.0
**Negative Inducements**	36.7	26.7
Jealousy Induction	0	0
Punish Infidelity Threat	6.7	0
Emotional Manipulation	16.7	3.3
Commitment Manipulation	10.0	3.3
Derogate Competitor	13.3	26.7
**Positive Inducements**	63.3	93.3*
Resource Display	3.3	20.0
Sexual Inducement	6.7	23.3
Appearance Enhancement	23.3	13.3
Love and Caring	36.7	33.3
Submission & Debasement	3.3	0
Direct Contact	46.7	76.7
Personality Advertisement	6.7	0
**Gain Access**	30.0	23.3
**Competitor Manipulation**	46.7	66.7
**Signal Possession**	26.7	46.7
Verbal Possession Signals	26.7	43.3
Physical Possession Signals	0	3.3
Possessive Ornamentation	0	0
**Negative Inducements**	33.3	46.7
Derogate Target	3.3	6.7
Threated Competitor	3.3	10.0
Violence against Rival	0	0
Direct Action	26.7	40.0
**Capitalize on Opportunity**	3.3	6.7
**Coalitional Strategy**	10.0	6.7
**Strategy Matching**	20.0	20.0

Global competitive strategies are bold, beneath which individual competitive tactics are listed.

* (*p* < .005) indicates a significant difference between the competitive strategies or tactics employed by competitors.

**Table 4:** Inter-sexual mate competition retention/poaching tactics in the Istmo Zapotec.

	Percentage Reported	
	Female Participant (*n* = 78)	*Muxe* Rival (*n* = 78)
**Target Manipulation**	66.7	96.2*
**Guarding**	33.3	2.6*
Direct Guarding	3.8	1.3
Vigilance	23.1	0*
Concealment	9.0	0
Monopolize	3.8	2.6
**Negative Inducements**	60.3	10.3*
Jealousy Induction	0	1.3
Punish Infidelity Threat	43.6	0*
Emotional Manipulation	23.1	0*
Commitment Manipulation	14.1	0*
Derogate Competitor	6.4	10.3
**Positive Inducements**	5.1	94.9*
Resource Display	0	23.1*
Sexual Inducement	1.3	43.6*
Appearance Enhancement	1.3	15.4*
Love and Caring	2.6	34.6*
Submission & Debasement	0	1.3
Direct Contact	2.6	62.8*
Personality Advertisement	0	1.3
**Gain Access**	1.3	35.9*
**Competitor Manipulation**	35.9	32.1
**Signal Possession**	24.4	16.7
Verbal Possession Signals	20.5	15.4
Physical Possession Signals	3.8	1.28
Possessive Ornamentation	0	0
**Negative Inducements**	17.9	24.4
Derogate Target	0	0
Threated Competitor	6.4	2.6
Violence against Rival	3.8	0
Direct Action	10.3	23.1
**Capitalize on Opportunity**	1.3	21.8*
**Coalitional Strategy**	3.8	0
**Strategy Matching**	3.8	2.6

Global competitive strategies are bold, beneath which individual competitive tactics are listed.

* (*p* < .005) indicates a significant difference between the competitive strategies or tactics employed by competitors.

## Results

### Frequency of inter-sexual mate competition

Among the 344 women in our Canadian sample, 52 (15.1%) had experienced inter-sexual mate competition. Because some women indicated that they experienced inter-sexual mate competition, but either declined to elaborate or provided too few details for meaningful analysis, there were 28 inter-sexual mate competition events detailed enough for analysis. Overall, 43% of Samoan women reported that they engaged in inter-sexual mate competition for a man with a *fa’afafine* rival, yielding 51 inter-sexual mate competition events detailed enough for analysis. Among women in the Istmo Zapotec, 85% reported that they engaged in inter-sexual mate competition for a man with a *muxe* rival, yielding 108 unique inter-sexual mate competition events (i.e., 8 women recounted only acquisition stories, 52 recounted only retention stories, and 24 women told independent stories detailing both acquisition and a retention competition). The frequency of inter-sexual mate competition significantly differed across cultures, *χ* ^2^ (2, *N* = 572) = 176.2, *p* < .001, and all pairwise comparisons were significant (Fisher’s Exact *p* < .001).

In all three cultures, inter-sexual competition occurred in which both competitors attempted to acquire a masculine male mate (Canada: *n* = 9; Samoa: *n* = 10; Istmo Zapotec: *n* = 30). However, the most common type of inter-sexual mate competition involved a woman attempting to retain her masculine male mate in the face of a male rival who endeavored to poach that mate (Canada: *n* = 19; Samoa: *n* = 41; Istmo Zapotec: *n* = 78).

### Features of Canadian inter-sexual mate competition events

Table 1 presents data for inter-sexual mate competitions in Canada. Because there were only 9 instances of inter-sexual mate competition for acquiring a mate, only data for mate poaching/retention interactions (*n* = 19) are presently reported. Although overall rates of most tactics tended to be low, both women and their gay male rivals directed their behaviours towards the target man, rather than toward each other. This involved gay men directing significantly more positive inducements, especially flirting, toward the target male, and women showing a suggestive difference in guarding behaviour (i.e., vigilance). In 47.4% of mate retention/poaching interactions, Canadian women reported engaging in no mate competition tactics whatsoever. This response underscores what many women conveyed qualitatively—target men were disinterested in same-sex contact and rebuffed male advances, and women did not perceive androphilic males as genuine rivals.

### Features of Samoan inter-sexual mate retention events

Table 2 presents data for inter-sexual mate competitions in Samoa, including participants’ reported behaviour, as well as that of the *fa’afafine* interloper. Because there were only 10 instances of inter-sexual mate competition for acquiring a mate, only data for mate poaching/retention interactions are reported. Participants directed the majority of their tactics toward their masculine male mate, using negative inducements as well as being vigilant against the advances of their rival. Conversely, *fa’afafine* focused most of their efforts on the target man, employing direct contact (flirting, etc.) as well as sexual inducements in a high number of competitive interactions. Women’s response to *fa’afafine* attempting to poach their mates was warranted, as *fa’afafine* successfully seduced their partners in 27% of reported retention/poaching events. One example involved a woman returning to her husband’s hospital room only to find the room vacant because a *fa’afafine* nurse had absconded with the man. Upon the eventual return of her husband, this woman insisted that he return home from the hospital, despite his reluctance to do so. Qualitatively, numerous women noted the intensity with which *fa’afafine* competed for the time and attention of target men. A 46-year-old woman, who was intimidated by a *fa’afafine* in a Samoan nightclub attempting to poach her mate, said, “when the *fa’afafine* get angry they are like lions—even with women. Heaps of *fa’afafine* give ladies a real hiding when they are angry.”

### Features of Istmo Zapotec inter-sexual mate acquisition events

Table 3 reports mate acquisition tactics in the Istmo Zapotec. Participants and their *muxe* competitors both directed most behaviour at the target male. *Muxe* were significantly more likely to direct positive inducements toward the target, including sexual provocations. Indeed, numerous female participants commented on *muxes*’ ability to engage in overtly sexual and flirtatious behaviours that would receive social censure if displayed by women. All individuals in these mate acquisition interactions primarily sought to impress the target male with positive inducements, although *muxe* employed these tactics more vigorously.

### Features of Istmo Zapotec inter-sexual mate retention events

Table 4 reports the frequency of tactics used during inter-sexual mate retention/poaching competitions. Within these interactions, participants’ behaviour tended to reliably differ from that of the *muxe* interloper. *Muxe* tended to employ tactics meant to entice the target male in some way (e.g., sexual inducements, flirtatious direct contact), and participants responded, in turn, by targeting their mate with negative inducements meant to secure their relationship. Despite the fact that *muxe* frequently engaged in overt sexual provocations of target men, 24.4% of female participants indicated that they did nothing in response to a *muxe* interloper because they believed that their partners were exclusively interested in women. Nevertheless, inter-sexual mate poaching interactions culminated with the *muxe* competitor having sex with the female rival’s masculine male mate 12% of the time. Some participants reported having reacted negatively during inter-sexual mate poaching attempts. A succinct qualitative illustration of this was given by a 27-year-old Zapotec woman:

“Yes, it was at a party that I was attending with my partner. A *muxe* came to talk to my partner. I overheard them talking, and I remember the *muxe* speaking very fondly to him. I got jealous. They stood up and walked away to be more distant. While it was happening, I would look at him very angry, as if saying ‘what’s going on there?’ He would look at me and just laugh, which made me even more upset. I didn’t say anything, but later I turned around and saw that they were kissing. I got mad and left the place. I never heard from them again, nor do I want to. The *muxe* knew that we came together.”

## Discussion

The present study documented the frequency and features of inter-sexual mate competition—females and males competing for the same romantic/sexual target—in three cultures. In Canada, where male bisexuality is relatively infrequent, such competition was rare, and features of inter-sexual mate competition events belie the fact that it was not taken seriously by many women. However, male bisexual behaviour is relatively more common in both Samoa and among the Istmo Zapotec, where masculine men regularly engage in sexual interactions with feminine *fa’afafine* and *muxe* respectively [16, 19]. In both cultures, women reported frequently engaging in inter-sexual mate competition against gender non-binary males, at rates that are appreciably higher than those found in Canada. It is possible that inter-sexual mate competition is more frequent in the West than was reported, but instances went unnoticed by Canadian women not anticipating such rivalry. Conversely, the Samoan and Istmo Zapotec data may over-estimate the true prevalence of inter-sexual mate competition, because women in these contexts are attuned to the possibility of competition with feminine *fa’afafine* and *muxe*, who are fully integrated in the culture.

Given that gender non-binary males represent 2–5% of the male population in both Samoa and the Istmo Zapotec [18, 36], it is perhaps surprising that 43% and 85% of women in each respective culture report having ever experienced inter-sexual mate competition. This seeming disparity highlights two important likelihoods. First, gender non-binary males are engaging in high degrees of mate acquisition behaviour, much of it in short-term contexts to acquire or mate-poach target men. Both *fa’afafine* and *muxe* seem to evidence a preference for short-term mating and a variety of novel sexual partners, a pattern consistent with the mating psychology/behaviour of Euro-American gay men [37–39]. Second, we did not ascertain the extent to which participant women competed with the same *fa’afafine* or *muxe*. Consequently, it is possible that a small absolute number of gender non-binary males are implicated in the high overall prevalence of inter-sexual mate competition. In other words, many participants may have engaged in mate competition against the same gender non-binary males. If so, the reported inter-sexual mate competition rivals may not be completely independent. Given that data were collected in numerous villages around the island of Upolu (Samoa), and the surrounding areas of Juchitán de Zaragoza (Oaxaca), we caution against over endorsement of this possibility. In any case, each mate competition event reported here represents an independent and unique interaction between a participant woman and a rival feminine male.

Despite the disparate cultural milieus in which these interactions took place, Samoan and Istmo Zapotec women employed similar strategies and tactics during inter-sexual mate poaching/retention interactions, as did *fa’afafine* and *muxes*. Women and gender non-binary males both attempted to manipulate the man who was the object of competition. They differed, however, in terms of the strategies they employed, given that female competitors were attempting to retain their masculine male mates, while gender non-binary male competitors were attempting to poach them. Samoan and Istmo Zapotec women were more likely to employ strategies such as Negative Inducements toward their mate (tactics: threat of retaliatory infidelity, emotional manipulation, demands for total commitment) and Mate Guarding (tactic: vigilance of mate). In contrast, *fa’afafine* and *muxe* competitors were more likely to employ strategies such as Positive Inducement (tactics: flirting, offers of sex) and Gaining Access (tactic: active attempts to interact with the masculine male target). Put simply, Samoan and Istmo Zapotec women were likely to engage in cost-inflicting mate retention behaviours, whereas feminine males employed benefit-provisioning poaching behaviours, mirroring the patterns observed in many intrasexual mate competitions in Euro-American cultures [33]. Gender non-binary males were also more likely to Capitalize on Opportunities that arose fortuitously, thereby facilitating attempts to entice masculine men in the absence of their female partners.

We do not expect that inter-sexual mate competition will be common in most species, or even in most human cultures. Nonetheless, when an appreciable number of individuals of one sex behave bisexually, then members of the opposite sex may be compelled to engage in inter-sexual mate competition to acquire and maintain reproductive partners when faced with rivals who are exclusively same-sex attracted. Depending on the species or the culture under consideration, one sex may behave in a relatively more bisexual manner than the other [40] and this will affect that manner in which inter-sexual mate competition is manifested. For example, inter-sexual mate competition for male sexual partners may be more prevalent in non-Western cultures where many masculine men behave bisexually, engaging in sexual interactions with both women and gender non-binary males [9, 15, 19, 23]. In contrast, inter-sexual mate competition for female sexual partners may be more prevalent in Western cultures, where female bisexual behaviour is relatively more common [41, 42], as is the case among the only other species for which data on inter-sexual mate competition exist, Japanese macaques [8].

All inter-sexual mate competition interactions involve a reproductive competitor and a non-reproductive one. Because we were interested in inter-sexual mate competition within the context of sexual selection, data collection focused on the experiences of reproductive competitors (i.e., Canadian, Samoan, and Istmo Zapotec women). Women’s mate competition narratives could be biased, however, inasmuch as participants presented themselves in a favorable light, or were absent when competitors employed certain tactics. In the future, a more comprehensive understanding of these interactions could be obtained by detailing the experiences of the non-reproductive competitors (i.e., gay men, *fa’afafine*, and *muxe*) as well, examining the possibility that women may attempt to poach the partners of same-sex attracted males. Additionally, the stories of inter-sexual mate competition comprising the present data set were undoubtedly richer and more complex than remembered and conveyed by participants, and the details put forth were then further stripped of information as behaviours were categorized using the taxonomy employed. Despite these limitations, some useful conclusions nonetheless emerged from the snapshot of inter-sexual mate competition provided by our female participants. Observational field-studies could help to fill in some of the potential gaps, although such research is difficult to conduct among humans, especially when the behavioural phenomena in question occur infrequently. It may also be possible to study inter-sexual mate competition in laboratory settings, examining women’s reaction to a male confederate who does or does not convey cues of engaging in inter-sexual mate competition.

Like intra-sexual competition for mates, inter-sexual mate competition involves strategies and tactics that can potentially influence the reproductive competitor’s access to reproductive partners. It is of great interest to determine whether inter-sexual mate competition involves uniquely evolved strategies, or if competitors simply co-opt strategies that are used in more commonly occurring intrasexual contests. In those species that engage in inter-sexual mate competition, our understanding of sexual selection and the evolution of mating systems may be improved by investigating the potential role that these interactions play in the acquisition and retention of reproductive partners. Such investigations will aid in developing a theoretical framework for understanding and making predictions about inter-sexual mate competition, moving beyond the basic description of the present research. Indeed, while much remains unknown regarding the *causes* of non-heterosexual attractions [11], future research should also investigate their social and sexual *consequences*, including the possibility that same-sex attractions and behaviours can act as a cultural and evolutionary force [43, 44] impacting the broader heterosexual mating market.

## References

[1] Janicke T, Häderer IK, Lajeunesse MJ, Anthes N. Darwinian sex roles confirmed across the animal kingdom. Science Advances. 2016;2(2):e1500983 Epub 2016/03/05. 10.1126/sciadv.1500983 26933680 PMC4758741

[2] Stockley P, Campbell A. Female competition and aggression: Interdisciplinary perspectives. Philos Trans R Soc Lond B Biol Sci. 2013;368(1631):20130073 Epub 2013/10/30. 10.1098/rstb.2013.0073 24167303 PMC3826202

[3] Barber N. On the relationship between country sex ratios and teen pregnancy rates: A replication. Cross-Cult Res. 2000;34(1):26–37. 10.1177/106939710003400102 WOS:000085135900002.

[4] Buss DM. The evolution of human intrasexual competition: Tactics of mate attraction. J Pers Soc Psychol. 1988;54(4):616–28. Epub 1988/04/01. 10.1037//0022-3514.54.4.616 . 3367282

[5] Buss DM, Shackelford TK, McKibbin WF. The Mate Retention Inventory-Short Form (MRI-SF). Personality and Individual Differences. 2008;44(1):322–34. 10.1016/j.paid.2007.08.013

[6] Brewer G, Hamilton V. Female mate retention, sexual orientation, and gender identity. Evolutionary Behavioral Sciences. 2014;8(1):12–9. 10.1037/h0097245

[7] Fisher M, Cox A. Four strategies used during intrasexual competition for mates. Personal Relationships. 2011;18(1):20–38. 10.1111/j.1475-6811.2010.01307.x

[8] Vasey PL. Female choice and inter-sexual competition for female sexual partners in Japanese macaques. Behaviour. 1998;135:579–97.

[9] Vasey PL, Leca JB, Gunst N, VanderLaan DP. Female homosexual behavior and inter-sexual mate competition in Japanese macaques: possible implications for sexual selection theory. Neurosci Biobehav Rev. 2014;46 Pt 4:573–8. Epub 2014/09/23. 10.1016/j.neubiorev.2014.09.002 . 25242104

[10] Murray SO. Homosexualities. Chicago, IL: The University of Chicago Press; 2000.

[11] Bailey JM, Vasey PL, Diamond LM, Breedlove SM, Vilain E, Epprecht M. Sexual Orientation, Controversy, and Science. Psychological Science in the Public Interest. 2016;17(2):45–101. Epub 2016/04/27. 10.1177/1529100616637616 . 27113562

[12] Nanda S. Gender Diversity: Crosscultural Variations. Long Grove, IL: Waveland Press; 2014.

[13] Whitam FL. Culturally universal aspects of male homosexual transvestites and transsexuals In: Bullough B, Bullough V, Elias J, editors. Gender Blending. Amherst, NY: Prometheus; 1997 p. 189–203.

[14] Vasey PL, VanderLaan DP. Evolving research on the evolution of male androphilia. The Canadian Journal of Human Sexuality. 2014;23(3):137–47. 10.3138/cjhs.23.3-CO1

[15] Vasey PL, VanderLaan DP. *Fa’afafine* In: Weekes-Shackelford V, Shackelford TK, Weekes-Shackelford VA, editors. Encyclopedia of Evolutionary Psychological Science. Cham: Springer International Publishing; 2016 p. 1–2.

[16] Mirandé A. Behind the Mask: Gender hybridity in a Zapotec community. Tucson, AZ: University of Arizona Press; 2017.

[17] Semenyna SW, VanderLaan DP, Petterson LJ, Vasey PL. Familial patterning and prevalence of male androphilia in Samoa. Journal of Sex Research. 2017;54(8):1077–84. Epub 2016/09/07. 10.1080/00224499.2016.1218416 . 27593894

[18] Gómez FR, Semenyna SW, Court L, Vasey PL. Familial patterning and prevalence of male androphilia among Istmo Zapotec men and muxes. PLoS One. 2018;13(2):e0192683 Epub 2018/02/22. 10.1371/journal.pone.0192683 29466410 PMC5821324

[19] Petterson LJ, Dixson BJ, Little AC, Vasey PL. Viewing time measures of sexual orientation in Samoan cisgender men who engage in sexual interactions with *fa'afafine*. PLoS One. 2015;10(2):e0116529 Epub 2015/02/14. 10.1371/journal.pone.0116529 25679961 PMC4332507

[20] Leser H. The Hirschfeld Institute of sexology In: Ellis A, Abarbanal AR, editors. Encylopedia of Sexual Behavior. New York: Hawthorne Books; 1961 p. 967–70.

[21] Whitam FL, Mathy RM. Male homosexuality in four societies: Brazil, Guatamala, the Philippines, and the United States. New York: Prager; 1986.

[22] Rahman Q, Xu Y, Lippa RA, Vasey PL. Prevalence of Sexual Orientation Across 28 Nations and Its Association with Gender Equality, Economic Development, and Individualism. Archives of Sexual Behavior. 2019 10.1007/s10508-019-01590-0 31797225 PMC7031179

[23] Whitam FL. Bayot and callboy: Homosexual-heterosexual relations in the philippines In: Murray SO, editor. Oceanic Homosexualities. New York, NY: Garland; 1992 p. 231–48.

[24] Fernández-Alemany M, Murray SO. Heterogendered homosexuality in Honduras. San Jose, CA: Writer’s Club Press; 2002.

[25] Vasey PL, Bartlett NH. What can the Samoan "Fa'afafine" teach us about the Western concept of gender identity disorder in childhood? Perspect Biol Med. 2007;50(4):481–90. Epub 2007/10/24. 10.1353/pbm.2007.0056 . 17951883

[26] Mirandé A. Hombres mujeres: An indigenous third gender. Men and Masculinities. 2016;19(4):384–409. 10.1177/1097184x15602746

[27] Devereaux G. Institutionalized Homosexuality of the Mohave Indians. Human Biology. 1937;9(498–527).

[28] Peacock JL. Rites of modernization: Symbolic and social aspects of Indonesian proletarian drama. Chicago: University of Chicago Press; 1968.

[29] Barbaro N, Shackelford TK, Weekes-Shackelford VA. Mothers and Fathers Perform More Mate Retention Behaviors than Individuals without Children. Hum Nat. 2016;27(3):316–33. Epub 2016/05/06. 10.1007/s12110-016-9261-z . 27147537

[30] Vanderlaan DP, Vasey PL. Mate retention behavior of men and women in heterosexual and homosexual relationships. Arch Sex Behav. 2008;37(4):572–85. Epub 2007/01/12. 10.1007/s10508-006-9139-y . 17216358

[31] Consulado de Carrera de México en Toronto. 2019 [Semptember 6, 2019]. Available from: https://consulmex.sre.gob.mx/toronto/index.php/en/servicesforeigners/visas?id=225.

[32] Kinsey AC, Pomeroy WB, Martin CE. Sexual behavior in the human male. Philadelphia, PA: Saunders; 1948. 10.2105/ajph.93.6.894 PMC1447861 12773346

[33] Pham MN, Barbaro N, Shackelford TK. Development and Initial Validation of the Coalitional Mate Retention Inventory. Evolutionary Psychological Science. 2014;1(1):4–12. 10.1007/s40806-014-0001-5

[34] GraphPad. Analyze a 2x2 contingency table: Graph Pad Software; 2018. Available from: https://www.graphpad.com/quickcalcs/contingency1.cfm.

[35] Benjamin DJ, Berger JO, Johannesson M, Nosek BA, Wagenmakers EJ, Berk R, et al Redefine statistical significance. Nature Human Behaviour. 2018;2(1):6–10. 10.1038/s41562-017-0189-z 30980045

[36] Semenyna SW, Petterson LJ, VanderLaan DP, Vasey PL. A Comparison of the reproductive output among the relatives of Samoan androphilic fa'afafine and gynephilic men. Archives of Sexual Behavior. 2017;46(1):87–93. Epub 2016/10/28. 10.1007/s10508-016-0765-8 . 27785648

[37] Bailey JM, Gaulin S, Agyei Y, Gladue BA. Effects of gender and sexual orientation on evolutionarily relevant aspects of human mating psychology. J Pers Soc Psychol. 1994;66(6):1081–93. Epub 1994/06/01. 10.1037//0022-3514.66.6.1081 . 8046578

[38] Lippa RA. The relation between sex drive and sexual attraction to men and women: a cross-national study of heterosexual, bisexual, and homosexual men and women. Archives of Sexual Behavior. 2007;36(2):209–22. Epub 2007/03/24. 10.1007/s10508-006-9146-z . 17380375

[39] Symons D. The evolution of human sexuality. New York: Oxford University Press; 1979.

[40] Goy RW, Goldfoot DA. Neuroendocrinology: Animal models and problems of human sexuality. Archives of Sexual Behavior. 1975;4:405–20. 10.1007/BF01541724 808193

[41] Gates G. How many people are Lesbian, Gay, Bisexual, and Transgender? 2011 Available from: https://williamsinstitute.law.ucla.edu/wp-content/uploads/Gates-How-Many-People-LGBT-Apr-2011.pdf.

[42] Geary RS, Tanton C, Erens B, Clifton S, Prah P, Wellings K, et al Sexual identity, attraction and behaviour in Britain: The implications of using different dimensions of sexual orientation to estimate the size of sexual minority populations and inform public health interventions. PLoS One. 2018;13(1):e0189607 Epub 2018/01/03. 10.1371/journal.pone.0189607 29293516 PMC5749676

[43] Bailey NW, Zuk M. Same-sex sexual behavior and evolution. Trends Ecol Evol. 2009;24(8):439–46. Epub 2009/06/23. 10.1016/j.tree.2009.03.014 . 19539396

[44] Vasey PL. Where do we go from here? Research on the evolution of homosexual behaviour in animals In: Sommer V, Vasey PL, editors. Homosexual behaviour in animals: An evolutionary perspective. Cambridge: Cambridge University Press; 2006.

